# Management of disseminated superficial actinic porokeratosis and intraepidermal squamous cell carcinoma with low‐dose radiation therapy

**DOI:** 10.1111/ajd.13605

**Published:** 2021-05-06

**Authors:** Fleur Kong, Tracy S Moreira‐Lucas, Artur Kaminski, Lynda Spelman

**Affiliations:** ^1^ Department of Dermatology Princess Alexandra Hospital Brisbane Queensland Australia; ^2^ GenesisCare Alexandria New South Wales Australia; ^3^ GenesisCare Wesley Medical Centre Auchenflower Queensland Australia; ^4^ Queensland Institute of Dermatology South Brisbane Queensland Australia

Dear Editor,

Porokeratosis is a chronic, progressive disorder of keratinisation characterised by small hyperkeratotic papules or annular plaques with elevated borders usually found on sun‐exposed areas such as the upper and lower extremities.^1^, ^2^ The most common form is disseminated superficial actinic porokeratosis (DSAP), which usually manifests in the fourth or fifth decade of life and is caused by various factors including ultraviolet radiation exposure, trauma, infection and immunosuppression.^3^ DSAP is more common in Australia due to high ultraviolet exposure, which drives the inherited tendency for DSAP to be fully expressed.^1^ DSAP can be difficult to treat because it is often recalcitrant to currently available treatments.^1^

We present a case of an 86‐year‐old male with multiple intraepidermal squamous cell carcinomas (IEC) and DSAP of both legs who was successfully treated with volumetric modulated arc therapy (VMAT), a modern form of radiation therapy. The patient presented with significant DSAP, IEC and hyperkeratotic actinic keratoses (AK) mainly affecting his bilateral lower legs (Fig. 1). The patient had previously been prescribed several therapies to treat his DSAP including cryotherapy, topical fluorouracil, compounded keratolytic cream (12% lactic acid, sodium pyrrolidone carboxylate and urea 10% cream), zinc impregnated stockings (when secondarily infected) and oral nicotinamide; however, none successfully slowed the progress of the disease. The patient had a history of a melanoma on his back, as well as multiple basal cell carcinoma (BCC) and squamous cell carcinoma (SCC). Due to his skin cancer history, the extent of his DSAP, and concurrent AKs and IECs, topical therapies had proven to be ineffective and surgical excision inappropriate because of the vast size of the area requiring treatment. The patient subsequently was referred to a radiation oncologist for consideration of radiation therapy.

**Figure 1:**
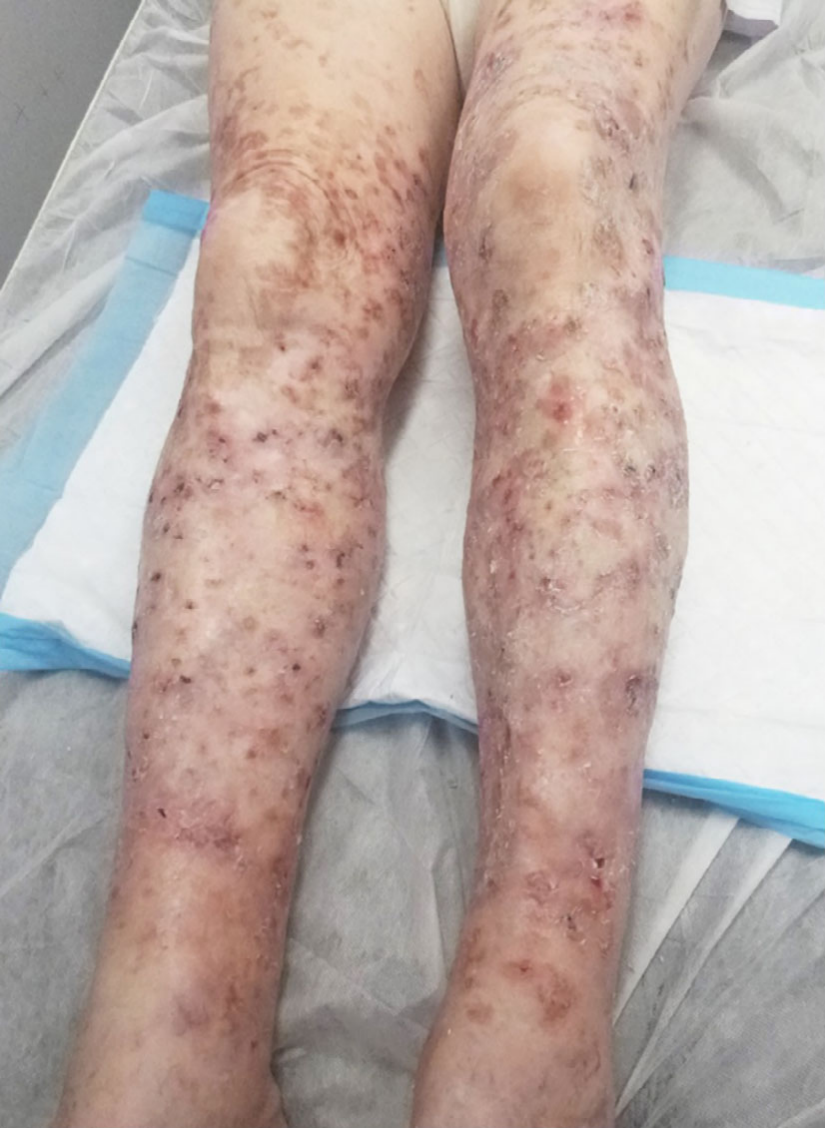
Pre‐treatment photograph of 86‐year‐old patient’s anterior bilateral legs with widespread DSAP and multiple intraepidermal squamous cell carcinomas.

Definitive radiation therapy was delivered using VMAT with 10 mm and 5 mm bolus on his right and left legs, respectively. Bolus is a material of a density similar to normal cutaneous tissue that is placed on the treatment area to influence radiation dose distribution. The patient’s prescribed treatment plan included 60 Gray in 30 fractions to the left leg and 54 Gray in 30 fractions to the right leg. However, as the patient was a full‐time carer for his wife, and after experiencing grade 2 radiation dermatitis on the anteromedial aspect of his left leg where he had previously sustained a hot steam injury, he opted to truncate his treatment. Thus, the patient only received a dose of 26 Gray delivered in 13 fractions over a two‐week period bilaterally to the legs.

After one week of treatment, the patient’s DSAP scales had begun to resolve, and at two weeks, there was a clinically significant improvement in DSAP. The patient was followed‐up by the radiation oncologist before being discharged back to his dermatologist for ongoing review. At two months post‐treatment, he had complete clinical resolution of his DSAP and IEC (Fig. 2). The patient continues to be followed‐up on an annual basis, and at four years post VMAT, he remains clear of IEC on both legs, although he has isolated DSAP lesions and some AK within the treatment field (Fig. 3).

**Figure 2:**
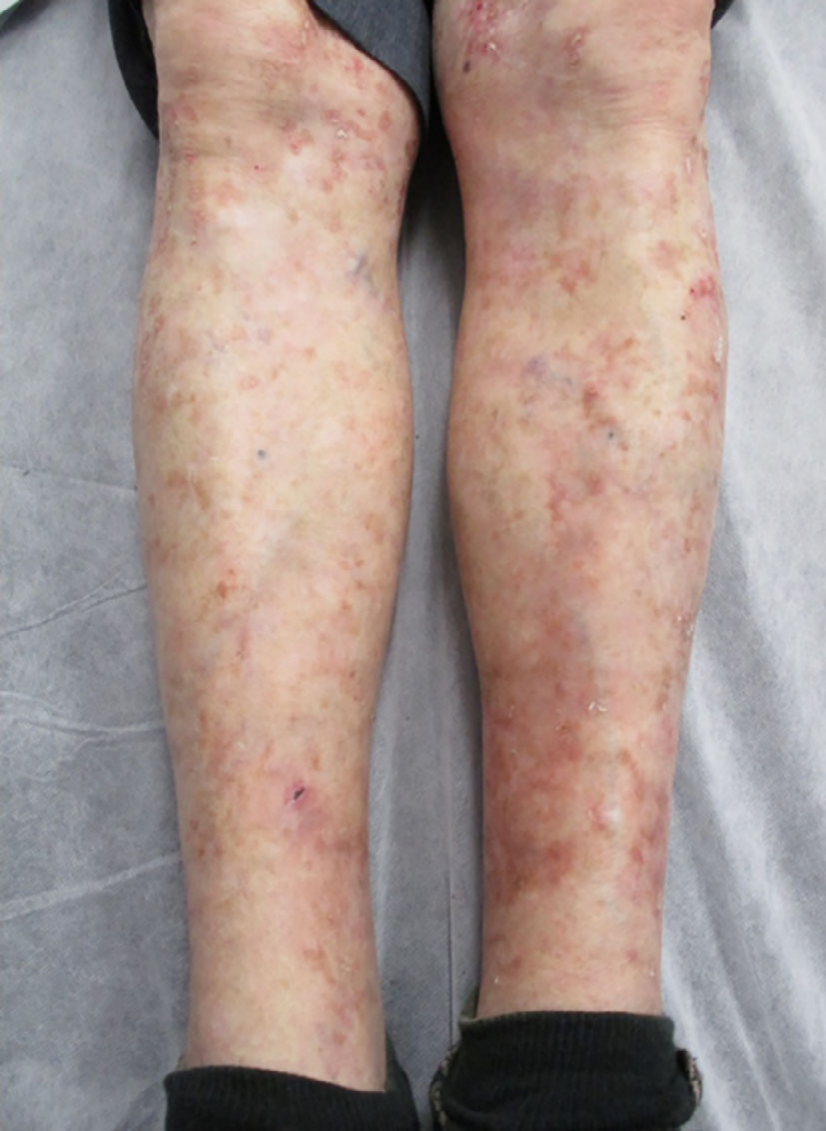
Anterior lower legs eight months post‐treatment. The patient experienced complete resolution of his DSAP, actinic keratosis and intraepidermal carcinoma in the treatment field.

**Figure 3:**
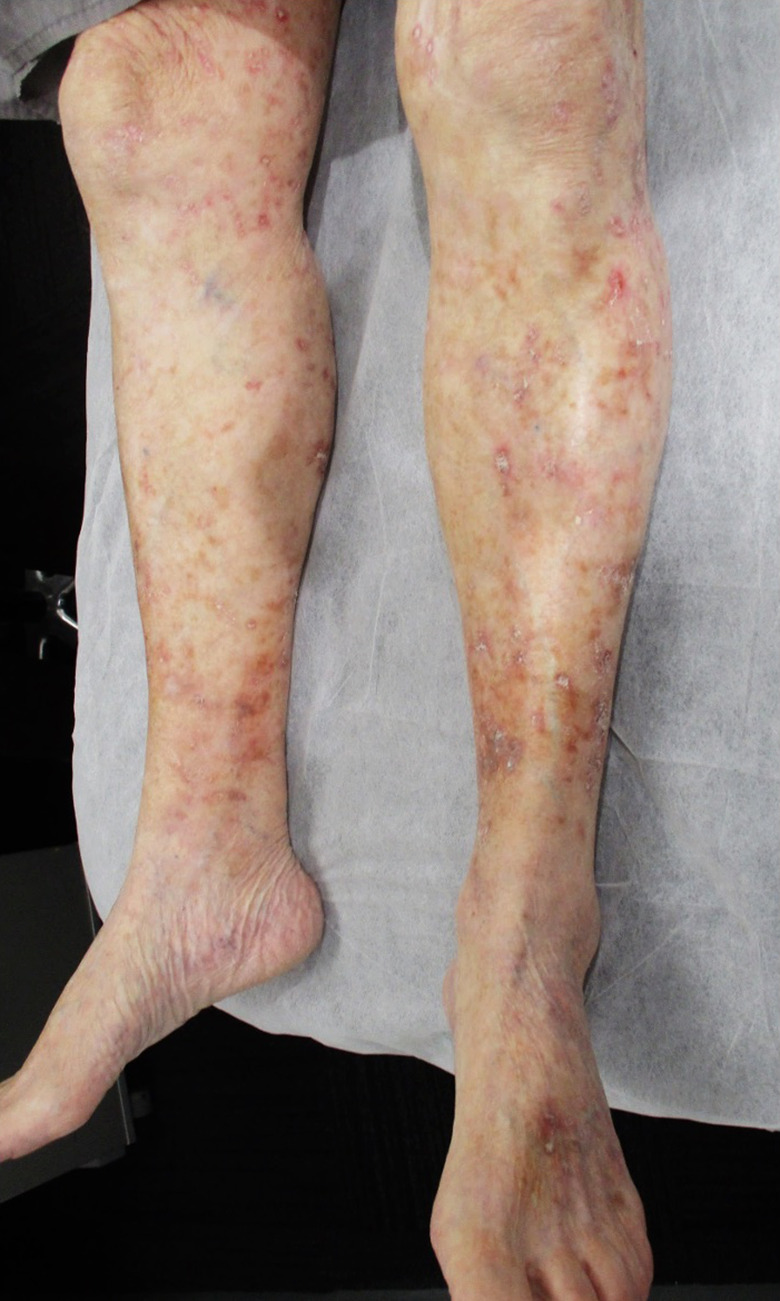
Anterior lower legs 2.5 years post‐treatment. The patient showed mild recurrence of his DSAP and actinic keratoses but remains clear of intraepidermal squamous cell carcinomas.

DSAP lesions are premalignant with conversion rates of 7.5 to 10%, most commonly to SCC; thus, there is a need to ensure that DSAP is treated effectively early in the disease course.^3^, ^4^ DSAP can be difficult to manage; however, treatment may include topical imiquimod and fluorouracil, vitamin D_3_ analogues, topical and oral retinoids, topical cholesterol/lovastatin, cryotherapy, excision, curettage, laser ablation, photodynamic therapy and radiation therapy.^1^, ^3^, ^5^, ^6^, ^7^, ^8^ The efficacy and long‐term durability of these treatments vary, and current data are primarily from case reports and case series.^1^, ^6^, ^7^, ^8^

Radiation therapy has been employed for decades to treat BCC and SCC, either definitively or as an adjuvant to surgical excision, in higher doses than what we report in this case.^9^, ^10^

Only one case series has reported on the successful use of radiation therapy in treating DSAP, using low‐energy Grenz rays.^5^ Thus, wide‐field VMAT with its capacity to be highly conformal and able to treat large, curved skin surfaces such as the legs and back with a homogenous dose may be a suitable option in complex DSAP with concomitant field cancerisation.

We thus report a case of successful treatment of extensive DSAP and concurrent non‐melanoma skin cancer in an elderly patient with low‐dose VMAT. Given these findings, we suggest that VMAT could be considered a suitable treatment option for patients with complex DSAP who have failed other treatments.

## References

[1] Ng DM, Brand R. A precancerous skin lesion that is often misdiagnosed. Aust. J. Gen. Pract. 2019; 48: 765–8. 31722456 10.31128/AJGP-04-19-4914

[2] Cohen BA. Papulosquamous Eruptions. In: Cohen BA (ed). Pediatric Dermatology (Fourth Edition). Baltimore, MD: W.B. Saunders, 2013.

[3] Le C, Bedocs PM. Disseminated Superficial Actinic Porokeratosis. [Updated 2020 Apr 30]. In: StatPearls [Internet]. Treasure Island (FL): StatPearls Publishing; 2020. Retrieved from: https://www.ncbi.nlm.nih.gov/books/NBK459202/ 29083728

[4] Ahmed A, Hivnor C. A case of genital porokeratosis and review of literature. Indian J. Dermatol. 2015; 60: 217. 10.4103/0019-5154.152587 PMC4372965 25814761

[5] Ramelyte E, Bylaite‐Bucinskiene M, Dummer R*et al*. Successful use of Grenz rays for disseminated superficial actinic porokeratosis: report of 8 cases. Dermatology 2017; 233: 217–22. 28817832 10.1159/000478855

[6] Skupsky H, Skupsky J, Goldenberg G. Disseminated superficial actinic porokeratosis: A treatment review. J. Dermatolog. Treat 2012; 23: 52–6. 20964569 10.3109/09546634.2010.495381

[7] Atzmony L, Lim YH, Hamilton C*et al*. Topical cholesterol/lovastatin for the treatment of porokeratosis: A pathogenesis‐directed therapy. J. Am. Acad. Dermatol. 2020; 82: 123–31. 10.1016/j.jaad.2019.08.043. 31449901 PMC7039698

[8] Weidner T, Illing T, Miguel D*et al*. Treatment of porokeratosis: A systematic review. Am. J. Clin. Dermatol. 2017; 18: 435–49. 28283894 10.1007/s40257-017-0271-3

[9] Likhacheva A, Awan M, Barker CA*et al*. Definitive and postoperative radiation therapy for basal and squamous cell cancers of the skin: executive summary of an American society for radiation oncology clinical practice guideline. Pract. Radiat. Oncol. 2020; 10: 8–20. 31831330 10.1016/j.prro.2019.10.014

[10] Cognetta AB, Howard BM, Heaton HP*et al*. Superficial x‐ray in the treatment of basal and squamous cell carcinomas: a viable option in select patients. J. Am. Acad. Dermatol. 2012; 67: 1235–41. 22818756 10.1016/j.jaad.2012.06.001

